# Web Thermo Tables – an On-Line Version of the TRC Thermodynamic Tables

**DOI:** 10.6028/jres.113.016

**Published:** 2008-08-01

**Authors:** Andrei Kazakov, Chris D Muzny, Robert D Chirico, Vladimir V Diky, Michael Frenkel

**Affiliations:** National Institute of Standards and Technology, Boulder, CO 80305-3328, USA

**Keywords:** database, thermophysical properties, web access

## Abstract

It has long been understood that availability of thermophysical and thermochemical property data is vital to scientific research and industrial design. For over 65 years, the Thermodynamics Research Center (TRC) has been publishing tables of critically evaluated data covering physical and thermodynamic properties of pure compounds, TRC Tables-Hydrocarbons and TRC Tables-Non-Hydrocarbons. Over their long history, the TRC Tables have always been valued as a reputable source of evaluated thermophysical and thermodynamic data. To facilitate more flexible, convenient, and up-to-date access to the data, here, we present the release of the on-line version of the TRC tables, Web Thermo Tables (WTT). Presently, WTT contains data for 7838 compounds and over 950,000 evaluated data points. The tabulated information includes critical properties, vapor pressures and boiling temperatures, phase transition properties, volumetric properties, heat capacities and derived properties, transport properties, reaction state-change properties, as well as index of refraction, surface tension, and speed of sound. Various search options and data plotting capabilities are provided via the Web interface. WTT are distributed through the NIST Standard Reference Data Program [1].

## 1. Introduction

On-demand availability of thermophysical and thermochemical property data has become essential to sustain rapid technological development in the modern world. To provide this availability, two major components have to be in place. First, an efficient information storage and retrieval mechanism must be established. In terms of current technology, this solution usually comes in the form of an electronic database. The second component is the efficient means of information delivery, and, in today’s world, it is indisputably the Internet. One of the best examples of this effective combination is the NIST Chemistry WebBook [2] that started in the mid-1990s and has proven to be very successful. However, for older, more “traditional” data compilations, the transition to electronic data delivery poses significant challenges. For any mature field (such as thermodynamics), significant amounts of data were collected and critically evaluated over many years, long before modern advances in information technology. Consequently, much of this information still exists in the form of “old” media, i.e., printed pages. Major efforts are being made today to bring printed information to an electronic form. One of the most visible examples of this process is an ongoing Google Books project [3,4] aimed to create a massive on-line library of scanned books. Processing with Optical Character Recognition (OCR) technology also allows indexing and, therefore, searches of scanned texts. Although scientific and engineering data represent a relatively small fraction of all printed information, the requirements on their digital delivery are disproportionately high. Merely having a scanned page with the table from a reference book is clearly not enough: the numerical data have to be reproduced very accurately and to have all associated metadata information (i.e., parameters and conditions uniquely defining the system) available for indexed searches.

The TRC Thermodynamic Tables project [5,6] is one of the oldest of its kind and has provided printed tables of high-quality, critically evaluated thermophysical and thermochemical data for over 65 years. Here, we describe a major effort to transfer the results of this project into the form of on-line accessible, searchable database, making this information readily and conveniently available to users worldwide.

## 2. An Overview of TRC Thermodynamic Tables

### 2.1 Brief History

The Thermodynamics Research Center (TRC) was founded in 1942 by Dr. Fredrick D. Rossini, Chief of the Section on Thermochemistry and Hydrocarbons at the National Bureau of Standards (NBS), to undertake American Petroleum Institute (API) Research Project 44. The purpose of that project was to obtain information on thermodynamic and thermophysical properties of selected hydrocarbons and their sulfur-containing derivatives. Such information was critically important to the development of new refinery technologies that were vital during World War II. Data tables were first circulated in loose-leaf sheets, and then were published by the Government Printing Office in bound book form, ca. 1948. “Selected Values of Physical and Thermodynamic Properties of Hydrocarbons and Related Compounds” comprising the tables of API-RP-44, extant as of December 31, 1952, were published for API by Carnegie Press [7]. The outstanding accomplishments of the staff of API Research Project 44 were readily apparent by the overwhelming acceptance of the work by industry and educational institutions worldwide. API Research Project 44 operated at NBS from its beginning in 1942 until 1950, when it moved to the Carnegie Institute of Technology (now Carnegie Mellon University), where Dr. Rossini was the Silliman Professor and Head of the Department of Chemistry.

In 1955, TRC started another national project – Manufacturing Chemists’ Association (MCA) (subsequently the Chemical Manufacturers Association, and now the American Chemistry Council) Research Project—at the Carnegie Institute of Technology. Its purpose was to expand coverage to all organic compounds, using the same kind of loose-leaf tables as API-RP-44. In 1961, TRC was relocated to Texas A&M University. Later, the project name was changed to the “Chemical Thermodynamic Properties Data Project” and the name of the tables was changed to “TRC Thermodynamic Tables—Hydrocarbons” and “TRC Thermodynamic Tables—Non-Hydrocarbons”. Shortly thereafter, the Thermodynamic Tables became self-supporting, and included spectral data sheets, which were part of the API-RP-44 and MCA projects from the beginning.

In September, 2000 TRC rejoined the National Institute of Standards and Technology as a part of the Physical and Chemical Properties Division. At NIST, the publication of both series of the TRC Thermodynamic Tables has continued to this day.

### 2.2 TRC Tables Update and Maintenance

Presently, the printed TRC Tables are updated quarterly. New or updated and/or corrected tables are prepared by data compilers selected worldwide among recognized experts with the corresponding areas of expertise. Each compiler is trained to follow a strict data evaluation protocol developed at TRC. The best effort is made to select the most accurate values available at the time of publication. Whenever possible, the numbers reported in the tables are based on experimental measurements, the results of which have been published in the scientific literature or have been obtained through personal communication with the investigator. When more than one source exists, the selected value may be taken from the source judged to be the most reliable. More often, however, the selected value is obtained by some additional evaluation—perhaps averaging, smoothing, or extrapolation of data from several sources. Older data may be corrected or recalculated using updated values of auxiliary data, fundamental constants, and/or conversion factors when deemed appropriate. In making the final choice, consideration is given not only to the directly measured property values, but also to other data related by thermodynamic principles to the data in question. Where experimental data are missing or unreliable, the data in the tables are obtained by a correlation or estimation procedure. Many correlations between thermodynamic properties and molecular structure and between changes in temperature and pressure are now known. Numerous correlations have been developed by TRC staff members during the past several decades. All correlations used for data evaluation are described as references or notes in the reference section.

Data compilers provide new tables in the form of specially formatted text files that, upon editing for the best uniform visual presentation, are converted into publication-ready TRC Table pages. Each update undergoes a review by the TRC Thermodynamic Tables Editorial Board and, if any problems are identified, they are either corrected on-site at TRC or communicated back to the compiler.

Recently, another layer of data verification has been added to this process, taking advantage of the newly developed [8,9] software package, TRC ThermoData Engine (TDE). TDE implements the dynamic data evaluation concept and produces critically evaluated data based on the experimental information derived from the TRC SOURCE database [10], a number of estimation methods, and enforcement of thermodynamic consistency among different properties for a given compound. If TDE is able to provide the evaluation for the data to be entered into the TRC Tables, a quantitative comparison is made, and if the differences between the evaluations by the TDE and a human compiler appear to be greater than the values of the uncertainties, further review of the data in question is conducted.

In the early 2000s, the process of conversion of the printed version of the TRC Tables into an electronic format (i.e., relational database) was begun. Because the overwhelming majority of the data existed only in printed copy (with some tables dating back to the TRC Tables origins, the API Project 44), the data were entered into the database manually. Later, a number of conversion software tools were developed to convert the compiler-provided text files into a format that can be used to load the data into the database directly. From that point, the updates of the electronic version of the TRC Tables occurred concurrently with the printed version.

### 2.3 Current Status of the TRC Tables

As of June 17, 2008, the TRC Tables contain 951,113 evaluated property points for 7838 pure compounds. A complete list of thermophysical and thermochemical properties provided in the TRC Tables is given in Table 1. When available, the properties are provided for different phases (i.e., different crystal forms, glass, liquid, real or ideal gas, and supercritical fluid) of a compound. In all cases, the estimated uncertainties in the tabulated values may be inferred from the number of significant figures used to display them. Not all properties listed in Table 1 have been evaluated for all compounds.

**Fig. 1:**
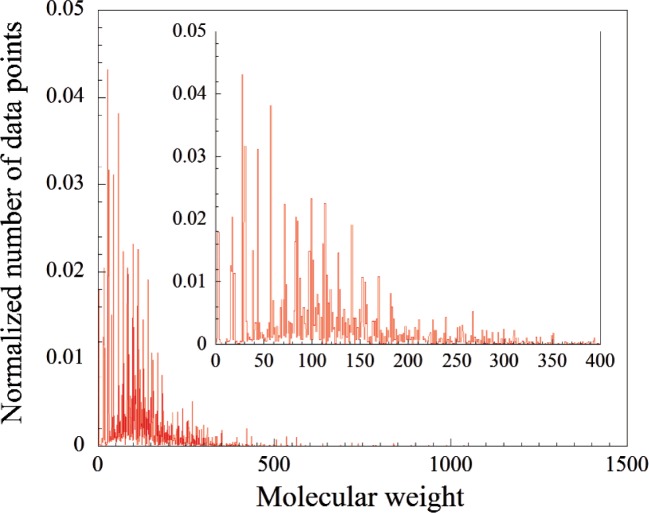
Statistical distribution of evaluated property values (data points) stored in the TRC Tables with respect to the molecular weight of the compound.

To further illustrate the availability of data, Fig. 1 shows a statistical distribution of the number of evaluated data points in the TRC Tables with respect to a compound’s molecular weight. As can be seen, the majority of data points correspond to compounds with molecular weights below about 200 amu. The distribution peaks at about 100 amu, with sharp discrete spikes observed over the 1-200 amu range, indicative of high data availability for a limited number of “popular”, well-studied compounds.

## 3. Development and Deployment of Web-Based TRC Tables

The objective of the present effort was to modernize the 65-year old TRC Tables project, which contains a wealth of valuable information compiled by several generations of experts. Many older datasets remain relevant even by today’s standards and needed no revisions, even though they were prepared in 1950s. To modernize the data delivery and to facilitate more flexible, convenient, and up-to-date access to the data, we developed an on-line version of the TRC tables, Web Thermo Tables (WTT).

### 3.1 Data Quality Assurance

Prior to development of the Web version and its deployment, the electronic version of the TRC Tables was investigated with regard to the presence of data errors. A number of significant data errors were identified that can be divided into three major categories:

1. “hard-copy” errors: these are the errors that were inevitably accumulated in the printed version of the TRC Tables over their long history and subsequently propagated into the electronic database. Older data, collected prior to introduction of modern review and validation procedures, are mainly affected by these errors;
2. manual entry errors: these errors affect the data that had to be entered manually (i.e., existed only in the printed version). These are primarily human errors such as typos, missed or misplaced values, erroneous unit conversions, etc.;
3. file conversion errors: these problems were caused by misinterpretation of the data files provided by compilers during their software conversion into a database-readable form. Every effort was made to assure that this conversion procedure would be as robust as possible; however, it became clear that some errors were almost unavoidable. The main reason is that the legacy formats used by data compilers were designed primarily to produce “visually-esthetic” printed tables to be read and interpreted by a human user, and the consistency necessary for parsing by a computer program was never of serious consideration. Large amounts of metadata needed for rigorous property definition are not stated explicitly in these files. In some cases, critical information is delivered via footnotes that could not be unambiguously interpreted by software. Finally, a large variety of different table formats, units, and data presentation approaches further complicates software-based interpretation.

Thorough data quality control measures have been taken to address these problems. Systematic error identification and correction were conducted using several approaches. As pointed out earlier, TDE software [8,9] has proven to be exceptionally useful in identifying data problems for those properties within its scope. However, only recently entered TRC Table data had been validated with TDE, as this utility was not available previously. During the present effort, validation against TDE results was conducted for all TRC Table data that lie within the scope of TDE. The validation was carried out by converting all data into ThermoML formatted files [11], which are fully supported by TDE. ThermoML is the IUPAC standard XML-based format for storage and exchange of experimental thermophysical and thermochemical property data.

In addition to the TDE error checks described above, data validation was also conducted using a number of software tools developed specifically for this project. The data that were tabulated as functions of an independent variable (for example, temperature) were checked for outliers that are usually indicative of typing errors. Whenever possible, different properties for a given compound were checked for thermodynamic consistency and satisfaction of fundamental relationships (for example, between heat capacity and enthalpy or between heat of combustion and the corresponding enthalpies of formation for reactants and products). Finally, an extensive manual review of the data was also performed.

The procedures outlined above were able to substantially improve the overall quality of the TRC Tables data. However, the issue of correctly loading new data using the compiler-provided data files still remained. An ideal solution might have been an adoption of a new, rigorously defined data file format to be used by the data compilers. Such a transition, however, would require changing the logistics of data preparation used by compilers as well as the preparation of the publication-ready TRC Table pages. Considering the amount of time it would take for development and retraining of personnel, this transition is planned to occur gradually in the near future, and an alternative, interim approach has been adopted. TRC has extensive experience with the massive collection of raw thermophysical property data; this experience has been translated into development of Guided Data Capture (GDC) software [12]. The GDC interface allows efficient processing of raw data by a human operator, while minimizing most common errors occurring during data collection by avoiding manual typing and unit conversion, enforcing known constraints, etc. A software package built on principles similar to those used in GDC has been developed specifically for TRC Table data collection directly from the final publication-ready tables. This approach provides the necessary human input for the data interpretation (inclusive of the interpretation of the information from the footnotes), while eliminating the majority of problems associated with manual data entry.

### 3.2 Development of Web Thermo Tables (WTT)

As for any Web data delivery system, WTT has two major components: an underlying database system and a Web interface.

From a database design point of view, the electronic version of the TRC Tables was not intended for direct access to the end users, but rather designed as an efficient and transparent storage solution. Therefore, a new, “presentation” database, derived from the original “storage” database, was developed for WTT. The main requirement for the presentation database design was the efficiency of information retrieval implemented via extensive indexing as well as some information redundancy. The resulting presentation database is running under the Oracle^1^ 10g engine, and its population and maintenance updates are automated via a series of scripts written in Oracle PL/SQL and Perl.

There are a number of promising modern technologies that provide full solutions for rich Web-based applications. For example, we have previously developed ILThermo, a web-accessible database for thermodynamic properties of ionic liquids [13] using the J2EE framework [14]. While offering a rapid development cycle and simplifying the codebase maintenance, this technology also adds substantial complexity and performance overhead to the resulting Web application. Considering the relative simplicity of WTT data structures and straightforward data presentation requirements, the Web interface for WTT was developed as a set of highly customized Common Gateway Interface (CGI) programs, optimized for overall efficiency and accessibility. Industry-standard HTML was used throughout the application. The usage of JavaScript was kept to a minimum and only as a means of convenient presentation. A user with no JavaScript support in a browser would still have full access to numerical data in tabular form. In fact, the numerical data can be accessed even from a text-based browser. An optional data plotting feature implemented with a Java applet [15] does require Java support in the user’s browser.

In addition to the efficiency and accessibility considerations described above, special emphasis has also been placed on providing an intuitive, visual, and simple interface, while avoiding unneeded distractions that are common in modern Web interface development.

**Fig. 2:**
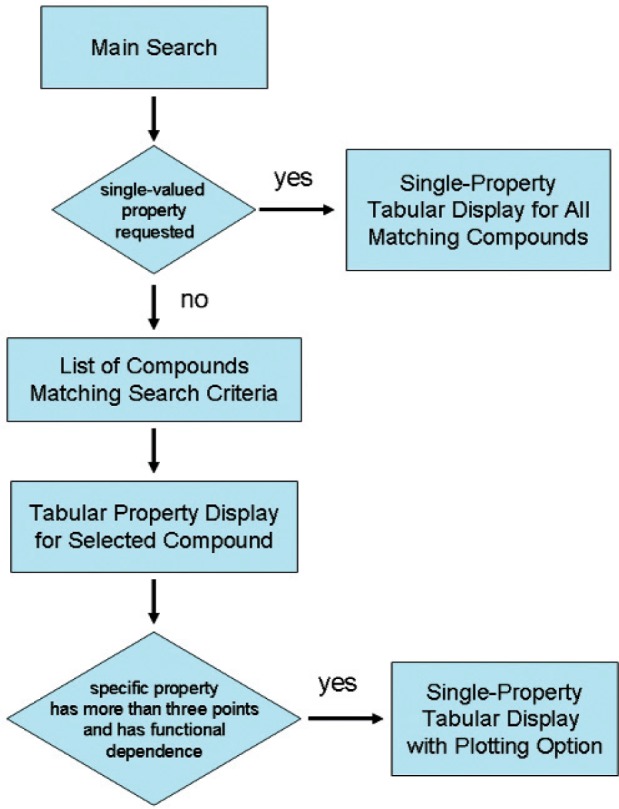
Schematic of WTT interface information flow.

**Fig. 3:**
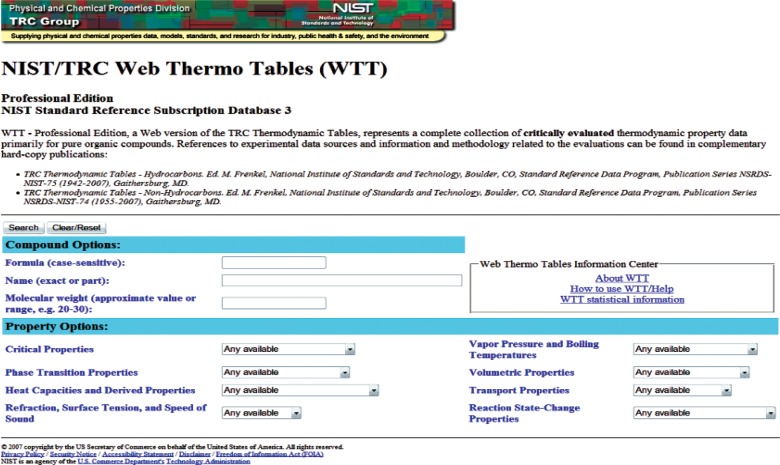
Main search page of the WTT interface.

A schematic of information flow for the WTT interface is depicted in Fig. 2. The user starts with the main search screen (Fig. 3) that allows data searches by compound and/or by property. Compound queries can be carried out by chemical formula, name (full or partial), and molecular weight. The property queries are grouped by property classes as listed in Table 1. Within each property class, the user can select specific property, “Any available”, or “None requested”. The choice of the latter would exclude all properties of the class from the search.

**Fig. 4:**
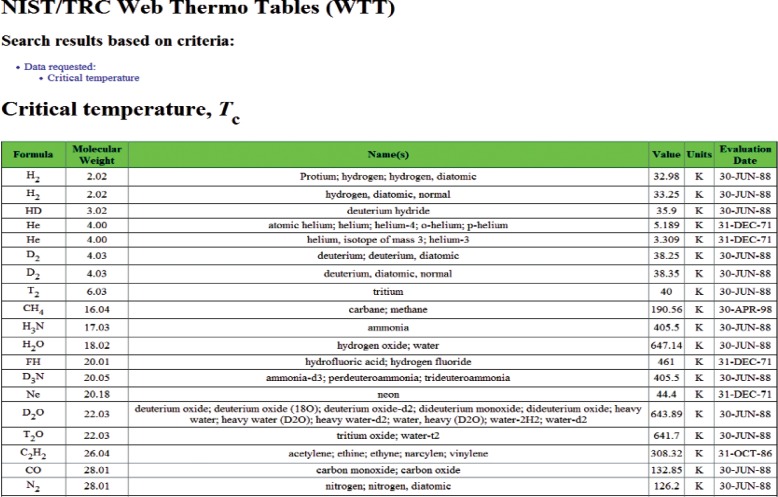
An example of data display for a single-valued property search (critical temperature).

**Fig. 5:**
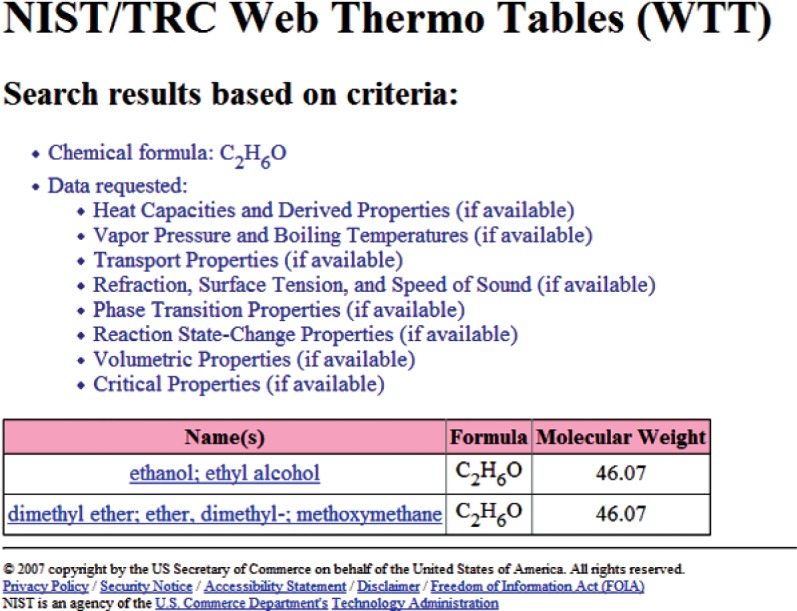
An example showing the result of compound search by chemical formula (“C2H6O”). A list consisting of two matching compounds (ethanol and dimethyl ether) is presented.

**Fig. 6:**
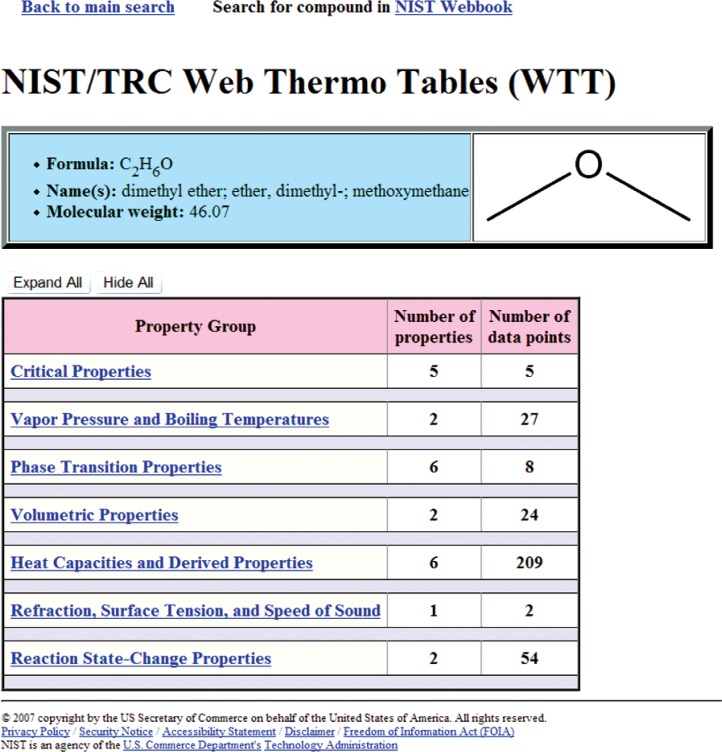
Tabular property display for a selected compound resulting from the selection of “dimethyl ether” for the case shown in Fig. 5.

**Fig. 7:**
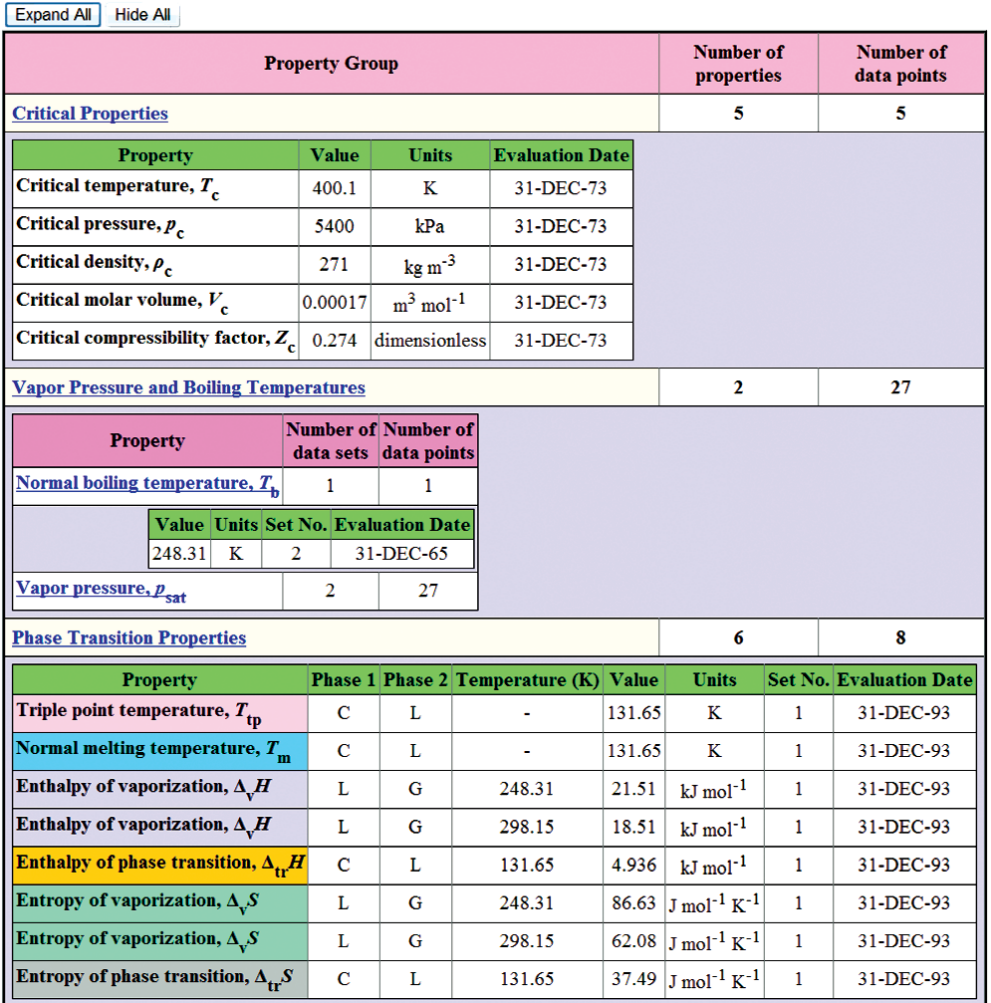
An example of expanding hierarchical panel structure for the case shown in Fig. 6.

**Fig. 8:**
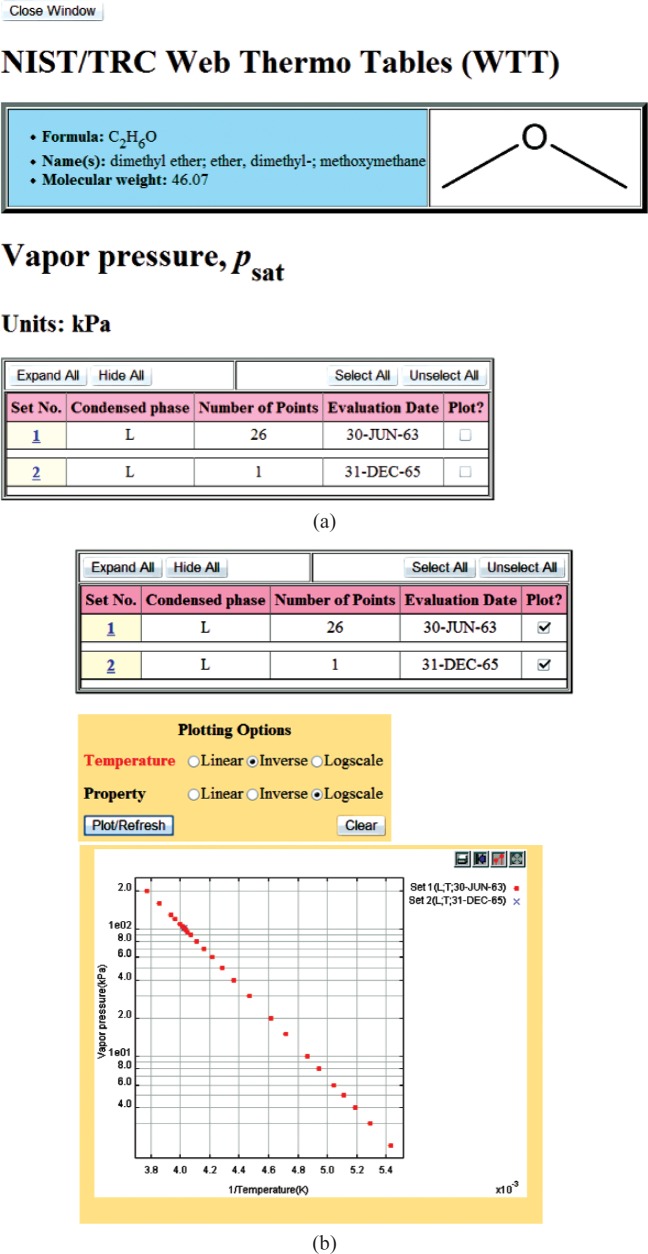
(a) An example of the specific property data presentation for a given compound when the data points are given as a function of an independent variable (temperature in this example); (b) An illustration of plotting capabilities for the case shown in Fig 8a.

Once the search query is submitted, the result (the next Web page) depends upon the properties chosen. If only one single-valued property is requested (presently, this includes any single property from the “Critical Properties” group and the normal boiling temperature) and all other property class searches are set to “None requested”, the final search result will be presented as a single table. An example of such search is shown in Fig. 4, where only critical temperature information was requested. Each row of the resulting table contains the description of the matching compound followed by the retrieved property value. In all other searches, the user is presented with the list of compounds matching the search criteria. An example of this page is shown in Fig. 5, where the search was conducted based on the chemical formula “C2H6O”, and two matching compounds, ethanol and dimethyl ether, were found. From this point, the user chooses the compound of interest and is taken to the next page, which presents a tabular property display for a selected compound (Fig. 6). Data presentation features an interactive hierarchical expanding/contracting panel structure (Fig. 7). The upper level lists available property groups and their statistical information (number of properties and total number of data points available). The next level down provides the list of specific properties and their statistics (number of datasets and total number of data points). If the total number of datapoints for a property is fewer than four, the table with the data is embedded on the lowest level (such as the normal boiling point example in Fig. 7). If the total number of datapoints for a property of interest is four or greater (such as the example for vapor pressure in Fig. 7), following the link for this property opens a new browser window (Fig. 8a) with a single-property tabular data display. Similar to the previous screen, the data are presented with an expanding/contracting panel structure. The upper level lists available datasets along with their phases and statistics, and the lower level contains the numerical data. Each dataset level also has a checkbox that can be checked if the user wishes to plot this dataset. If one or more checkboxes are selected, the “Plotting Options” menu is activated below (Fig. 8b) that gives user three choices for each axis (linear, inverse, or log). Clicking on the “Plot/Refresh” button will activate the Java plotting applet with the resulting data plot appearing below (Fig. 8b). If selected datasets are tabulated as functions of two variables, two independent plotting panels will be provided, one for each variable.

As mentioned previously, the interactive part of the Web interface is driven entirely by a very compact JavaScript code embedded directly into the HTML page. If JavaScript functionality is absent or disabled in the user’s browser, all panels will appear expanded, and direct access to the numerical data tables is still available.

### 3.3 Deployment of WTT

WTT was deployed on the public NIST server at the end of 2007. Access to the data is available via the NIST Standard Reference Data Program. Public release of WTT is available in two editions: Professional and Lite. The Professional edition contains the complete TRC Tables collection, while the Lite edition restricts the data to those for 150 common (primarily organic) compounds. Complimentary WTT data availability information (specific properties for a given compound, parameter ranges, number of points) can be accessed on-line (http://wtt-pro.nist.gov and http://wtt-lite.nist.gov for the Professional and Lite editions, respectively).

## 4. Summary

A new, Web-based version of the TRC Thermodynamic Tables, Web Thermo Tables (WTT), has been developed. The electronic version of the Tables (i.e., the underlying relational database) has gone through systematic data quality-control measures that resulted in correction of numerous data errors accumulated over the course of the TRC Tables’ long history. The final product features a simple, visual, and intuitive interface that has been optimized for the efficient retrieval of information. Interactive data plotting capabilities are also provided. This new NIST product has been released in two editions: Professional (the entire data collection) and Lite (data for 150 selected commonly used compounds). User access to WTT editions is available via the NIST Standard Reference Data Program.

## Figures and Tables

**Table 1:** List of thermophysical properties provided in the TRC Tables

Property Group	Property
Critical Properties	Critical temperature
	Critical pressure
	Critical density
	Critical molar volume
	Critical compressibility factor
Vapor Pressure and Boiling Temperatures	Normal boiling temperature Vapor pressure
Phase Transition Properties	Triple point temperature
	Normal melting temperature
	Enthalpy of vaporization
	Enthalpy of phase transition
	Entropy of vaporization
	Entropy of phase transition
Volumetric Properties	Specific density
	Adiabatic compressibility
	Compressibility factor
	Second virial coefficient
Heat Capacities and Derived Properties	Heat capacity at constant pressure
	Heat capacity at saturation
	Enthalpy
	Entropy
	Enthalpy function
	Gibbs energy function
Transport Properties	Viscosity
	Kinematic viscosity
	Thermal conductivity
Refraction, Surface Tension, and Speed of Sound	Surface tension
	Speed of sound
	Refractive index
Reaction State-Change Properties	Enthalpy of formation
	Gibbs energy of formation
	Enthalpy of combustion (gross)
	Enthalpy of combustion (net)

## Footnotes

1 Certain commercial equipment, instruments, or materials are identified in this paper to foster understanding. Such identification does not imply recommendation or endorsement by the National Institute of Standards and Technology, nor does it imply that the materials or equipment identified are necessarily the best available for the purpose.
